# Optimizing the preparation of thin endometria in frozen-thawed embryo transfer: a retrospective cohort analysis

**DOI:** 10.3389/fendo.2025.1490092

**Published:** 2025-10-01

**Authors:** Xiaoxue Ji, Chunxiao Wei, Zhiru Liu, Lijun Qiu, Jianwei Zhang

**Affiliations:** ^1^ The First Clinical College, Shandong University of Traditional Chinese Medicine, Jinan, Shandong, China; ^2^ Reproductive Center of Integrated Medicine, Shandong University of Traditional Chinese Medicine Affiliated Hospital, Jinan, Shandong, China

**Keywords:** endometrial preparation, thin endometrium, pregnancy outcome, frozen-thawed embryo transfer, hormone replacement therapy, natural cycle

## Abstract

**Background:**

To date, there is no consensus on the optimal endometrial preparation protocol for frozen-thawed embryo transfer (FET) in patients with a thin endometrium. This study evaluated the effects of different endometrial preparation protocols on pregnancy outcomes in patients undergoing FET cycles.

**Methods:**

In this retrospective cohort analysis, we included women with a thin endometrium who underwent FET cycles at the Department of Gynaecology, Affiliated Hospital of Shandong University of Traditional Chinese Medicine, from January 1, 2015, to November 30, 2023. Based on the endometrial preparation protocols, the participants were divided into two groups: natural cycle (NC) and hormone replacement therapy (HRT). The primary outcomes measured were clinical pregnancy and live birth rates. Propensity score matching (PSM) was used to mitigate potential disparities between the groups. A comparative analysis of pregnancy outcomes was then performed between the groups.

**Results:**

No statistically significant differences were found in pregnancy outcomes between the two groups, even after applying PSM. However, patients with an endometrial thickness of ≤7 mm on the trigger day exhibited significantly higher rates of clinical and biochemical pregnancies when assigned to the HRT group.

**Conclusions:**

The HRT protocol is advisable for FET cycles in patients with thin endometrium, particularly when the endometrial thickness is <7 mm on the day of hormonal trigger administration.

## 1 Background

As reproductive technology advances, the practice of frozen-thawed embryo transfer (FET), which involves cryopreserving embryos remaining after *in vitro* fertilization (IVF) or those unsuitable for immediate transfer during ovulation induction, has gained significant traction (1). These embryos can be preserved for future use, thus avoiding waste, reducing costs, saving time, increasing the cumulative pregnancy rate, and reducing the occurrence of ovarian hyperstimulation (2, 3). FET efficacy depends on several factors, including embryo quality, endometrial tolerance, and the degree of synchronization between the embryo and the endometrium (4). Once embryo quality is ascertained, meticulous endometrial preparation becomes crucial in determining endometrial receptivity, a critical determinant of pregnancy outcomes (5). Endometrial thickness and morphology are commonly assessed via transvaginal ultrasonography, which provides an initial measure of endometrial receptivity.

A thin endometrium (TE) is defined as an endometrial thickness below the threshold for successful embryo implantation. However, there is currently no standardized criterion for defining this threshold, and one study (6, 7) demonstrated that the prevalence of TE in patients undergoing assisted reproductive technology for pregnancy was 2.4%. In 2019, the Canadian Fertility & Andrology Society published guidelines (8) defining a TE as an endometrial thickness (EMT) of <8 mm on the trigger day and <7 mm in an FET cycle. These guidelines recommend canceling the current cycle transfer in such cases. Similarly, the European Society of Human Reproduction and Embryology published guidelines advising that patients with an EMT <8 mm on the trigger day or egg collection may be at risk of reduced pregnancy rates. Several studies have demonstrated that an EMT of 8–14 mm is optimal for embryo implantation (9). A TE impairs endometrial tolerance, reduces the rate of embryo implantation, and results in suboptimal pregnancy outcomes (10).

Although the pathogenesis of a TE is not fully understood, research suggests a link between deficiencies in estrogen and its receptors (11, 12). Hormone replacement therapy (HRT) promotes the proliferation of endometrial epithelial cells through the exogenous administration of estrogen and progesterone, thus improving endometrial tolerance. Consequently, using HRT protocol to enhance endometrial growth in patients with a TE is the optimal approach for FET. This approach allows the sufficient induction of an increase in the number of estrogen receptors in the endometrium, thereby maximizing the application of exogenous estrogen to the endometrium. This, in turn, facilitates an increase in EMT to the greatest extent possible.

However, previous studies have demonstrated that endogenous estrogen is more favorable for increasing endometrial tolerance. Furthermore, protocol is associated with a higher rate of early pregnancy loss (13) and a higher risk of pregnancy hypertensive disorders (14–16) than natural cycles (NCs). Additionally, older women have a higher incidence of pregnancy complications than women of appropriate age (14, 17), and most cases of TE occur in older women (18). This necessitates further research to identify the most appropriate endometrial preparation program.

Therefore, in this study, we aimed to evaluate the effectiveness of various endometrial preparation protocols in improving pregnancy outcomes for patients with a TE undergoing FET. Specifically, we aim to compare hormone replacement cycles with natural cycles, investigating their impact on EMT, implantation rates, and overall pregnancy success. The findings of this study have the potential to significantly enhance clinical practices in reproductive medicine, offering optimized treatment strategies that can improve the success rates of FET and contribute to better reproductive health outcomes for patients.

## 2 Materials and methods

### 2.1 Study design

This retrospective cohort analysis included women with TE who underwent FET cycles. The participants received treatment at the Department of Gynaecology, Affiliated Hospital of Shandong University of Traditional Chinese Medicine, from January 1, 2015, to November 30, 2023. The study divided participants into two groups based on different endometrial preparation protocols: the NC and the HRT groups. We analyzed both groups regarding their baseline clinical characteristics and pregnancy outcomes. PSM was applied to minimize potential differences between groups, accounting for baseline characteristics and confounding factors. Due to the retrospective nature of this study, the requirement for informed consent was waived. The study design was approved by the institutional review board (2024-086-KY) of Shandong University of Traditional Chinese Medicine Affiliated Hospital.

### 2.2 Patient inclusion

The inclusion criteria were as follows: (1) a trigger EMT of 6–8 mm in previous stimulation cycles, and (2) an age of 40 years or younger at the time of *in vitro* fertilization (IVF). Exclusion criteria included the presence of uterine adhesions, endometrial polyps, advanced endometriosis (stage IV), adenomyosis, and any uterine anomalies.

### 2.3 Endometrial preparation protocols

The endometrial preparation protocols used in this study included NC and HRT. Clinicians selected the most suitable protocol based on the patients’ conditions and their clinical experience; however, no rigorous standardized guidelines in clinical practice exist for this condition.

#### 2.3.1 FET with NC protocol

The NC protocol was suitable for women with regular menstrual cycles, and a modified NC protocol is typically used. Between days 8 and 10 of the menstrual cycle, transvaginal ultrasound was performed to assess follicular size, EMT, and morphology. When the follicle reached a diameter of 12–14 mm, oral estradiol valerate (2–4 mg/day) was initiated and adjusted based on EMT and follicular development. At a follicle diameter of 18 mm, serum hormone levels were used to determine the timing of ovulation. Human chorionic gonadotropin (hCG) (2000–10000 IU) was administered intramuscularly to trigger ovulation. Progesterone (20 mg/day) was administered intramuscularly along with oral dydrogesterone (20 mg/day) to facilitate endometrial maturation.

#### 2.3.2 FET with HRT protocol

Oral estradiol valerate (4 mg/day) was initiated on days 2–3 of the menstrual cycle or following withdrawal bleeding due to hormonal medications. Transvaginal ultrasound was used to assess the EMT, and the estradiol valerate dosage was tailored based on endometrial measurements and serum hormone levels, not exceeding a maximum of 8 mg/day. When the EMT reached 8 mm or greater, intramuscular progesterone injections (20 mg/day) were administered concurrently with oral dydrogesterone (20 mg/day) to facilitate endometrial transformation.

### 2.4 Measurement of endometrial thickness

In this study, a GE Voluson E8 color Doppler ultrasound diagnostic system was employed, with the probe frequency set at 5.0-9.0 MHz. All ultrasound examinations were conducted by the same experienced operator to ensure consistency and accuracy of measurements, Patients were instructed to empty their bladders prior to the examination and were positioned in the lithotomy position, Using transvaginal two-dimensional ultrasound imaging, the thickest portion of the endometrium was identified and measured. To minimize measurement error, three separate measurements were taken for each patient, and the mean value was calculated and recorded as the final endometrial thickness on the trigger day.

### 2.5 Outcomes

The primary outcomes of this study were clinical pregnancy and live birth rates. Secondary outcomes were biochemical pregnancy and ectopic pregnancy rates. Clinical pregnancy was identified by ultrasound detection of at least one gestational sac within the uterine cavity approximately 28 days after embryo transfer. Live birth was defined as the delivery of at least one living fetus. Biochemical pregnancy was identified as having a serum β-HCG level >5 mIU/mL approximately 14 days post-embryo transfer. Ectopic pregnancy was characterized by embryonic development outside the uterine cavity.

### 2.6 Statistical analyses

Statistical analyses were conducted using IBM SPSS Statistics for Windows, version 26.0 (IBM Corp., Armonk, NY, USA). Continuous variables adhering to a normal distribution were reported as the mean ± standard deviation and analyzed using the Chi-square test; missing values were imputed with series means. For variables that did not adhere to a normal distribution, the median (interquartile range) was reported, and missing values were imputed with the median. One-way analysis of variance (ANOVA) was used to compare data that conformed to a normal distribution, whereas the Kruskal–Wallis test was applied for non-normally distributed data. Categorical data are presented as frequencies and percentages and were compared across groups using the Chi-square test or Fisher’s exact test when appropriate. Logistic regression analysis was performed to adjust for confounding factors. Subsequently, 1:1 matching was performed using PSM with a caliper width of 0.02, and propensity scores were derived from logistic regression models that included baseline characteristics and variables that potentially influenced pregnancy outcomes. Subgroup analyses were also performed, which were adjusted for the duration of infertility, body mass index, baseline hormonal profile, type of embryos, number of embryos, and number of good-quality embryos before PSM; forest plots were created using R software version 4.3.2. Statistical significance was set at p < 0.05.

## 3 Results

### 3.1 Baseline and cycle characteristics

According to the inclusion and exclusion criteria, a total of 448 FET cycles were analyzed in patients with a TE. Of these, 256 were in the HRT group, and 192 were in the NC group. The baseline characteristics of the patients are detailed in **Table 1**. Before PSM, differences in the basal FSH levels between the two groups were statistically significant (p < 0.05). After PSM, there were no statistically significant differences between the two groups regarding maternal age at FET, BMI, duration of infertility, type of infertility, type and number of embryos, and number of good-quality embryos. Good-quality embryos were defined as those graded 2 or higher with 6–10 cleavage globules on the third day after egg retrieval or embryos graded BB or higher on the fifth day.

**Table 1:** General characteristics of patients with different endometrial preparation protocols.

		Before PSM			After PSM		
		NC	HRT	P value	NC	HRT	P value
Cases		263	326		236	236	
Maternal age at FET, years		35 (32, 38)	34 (31, 37)	0.01^a^	34 (32, 37)	35 (31, 38)	0.94
Duration of infertility, years		3 (2, 4)	3 (2, 4)	0.55	3 (2, 4)	2 (2, 4)	0.28
Body mass index, kg/m²		22.6 (20.5, 24.8)	22.7 (20.5, 25)	0.39	22.5 (20.5, 24.8)	22.5 (20.3, 24.5)	0.88
Type of infertility				0.11			0.76
	Primary infertility, n (%)	25.9% (68/263)	31.9% (104/326)		27.5% (65/236)	28.8% (68/236)	
	Secondary infertility, n (%)	74.1% (195/263)	68.1% (222/326)		72.5% (171/236)	71.2% (168/236)	
Baseline hormonal profile	FSH, mIU/mL	8.39 (6.55, 10.26)	7.92 (6.33, 9.61)	0.01^a^	8.31 (6.42, 9.91)	8.16 (6.37, 9.69)	0.33
	LH, mIU/mL	4.13 (3.09, 5.7)	4.37 (3.19, 6.39)	0.09	4.17 (3.09, 5.79)	4.12 (2.96, 5.94)	0.87
	E2, pg/mL	42 (31, 62)	43 (32, 58.93)	0.94	42 (31, 59)	43.5 (33.13, 59)	0.89
	P, ng/mL	0.52 (0.31, 0.83)	0.67 (0.32, 0.83)	0.03^a^	0.54 (0.33, 0.83)	0.55 (0.29, 0.77)	0.91
Type of embryos				0.32			0.76
	Cleavage embryo	90.9% (239/263)	9.1% (24/263)		90.3% (213/236)	9.7% (23/236)	
	Blastocyst	88.3% (288/326)	11.7% (38/263)		89.4% (211/236)	10.6% (25/236)	
Number of embryos		2 (1, 2)	2 (1, 2)	0.58	2 (1, 2)	2 (1, 2)	0.98
Number of good-quality embryos		2 (1, 2)	2 (1, 2)	0.56	2 (1, 2)	2 (1, 2)	0.84

p < 0.05 was considered statistically significant. ^a^Statistically significant.

NC, Natural Cycle; HRT, Hormone Replacement Therapy; PSM, Propensity Score Matching; FET, Frozen Embryo Transfer; FSH, Follicle-Stimulating Hormone; LH, Luteinizing Hormone; E2, estradiol; P, Progesterone; n, Number (used to denote counts); %, Percentage.

### 3.2 PSM

A total of 372 cycles were included for PSM. There were no statistically significant differences between the two groups in terms of baseline characteristics or other variables (**Table 1**). Pregnancy outcomes also did not differ significantly between the groups, as shown in **Table 2**.

**Table 2:** Clinical outcomes between the natural cycle and hormone replacement therapy protocols after propensity score matching.

Outcome	OR (95% CI)	P value
Clinical pregnancy rate	1.36 (0.91, 2.03)	0.129
Live birth rate	1.11 (0.71, 1.71)	0.655
Biochemical pregnancy rate	1.32 (0.89, 1.95)	0.164
Ectopic pregnancy rate	1.00 (0.20, 5.01)	>0.99

OR, Odds Ratio; CI, Confidence Interval.

### 3.3 Pregnancy outcomes

The pregnancy outcomes are presented in **Table 3**. After adjusting for age, BMI, duration of infertility, type of infertility, type of embryo, number of embryos transferred, and number of good-quality embryos transferred using multifactorial logistic regression, the clinical pregnancy rate, live birth rate, and biochemical pregnancy rate were higher in the HRT group compared to the NC group. The ectopic pregnancy rate was lower in the HRT group, but this difference was not statistically significant.

**Table 3:** Adjusted odds ratios of the outcomes between natural cycle and hormone replacement therapy after pre-propensity score matching.

Outcome	OR (95% CI)	P value
Clinical pregnancy rate	1.27 (0.87, 1.87)	0.216
Live birth rate	0.92 (0.60, 1.42)	0.72
Biochemical pregnancy rate	1.23 (0.84, 1.80)	0.286
Ectopic pregnancy rate	0.79 (0.15, 4.19)	0.778

Adjusted for maternal age at FET, duration of infertility, body mass index, type of infertility, baseline hormonal profile, type of embryos, number of embryos, and number of good-quality embryos.

OR, Odds Ratio; CI, Confidence Interval.

### 3.4 Subgroup analysis

**Figure 1:**
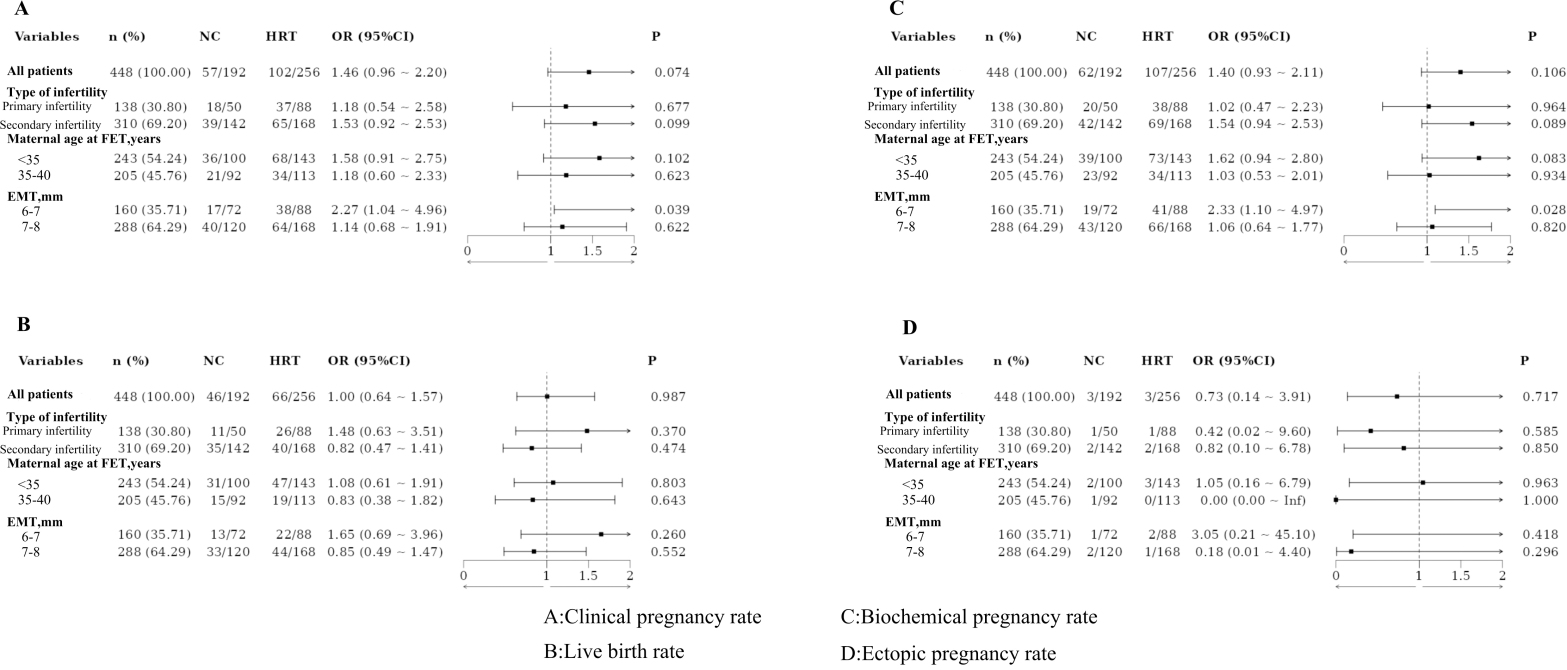
Adjusted odds ratios for subgroup analysis before propensity score matching.

**Figure 2:**
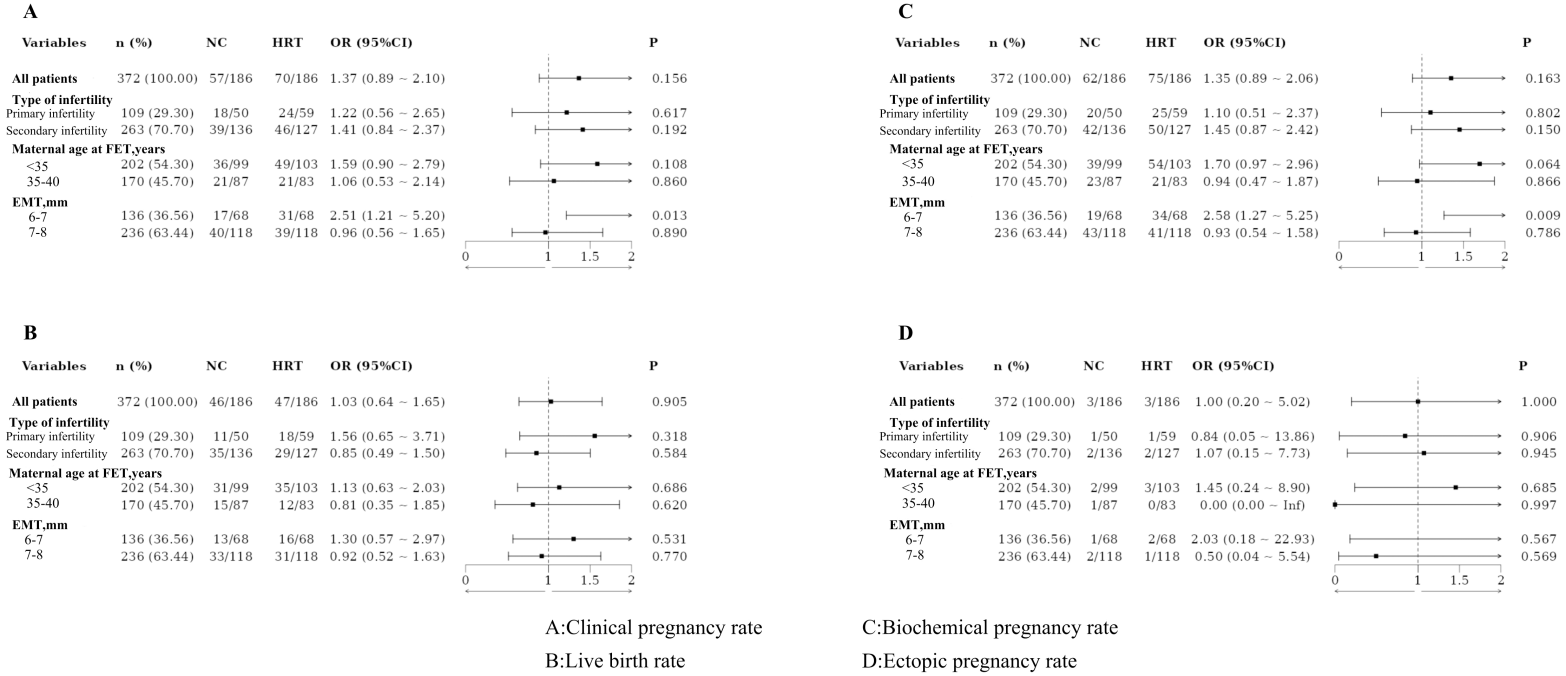
Forest plot for subgroup analysis after propensity score matching.

Based on the type of infertility, the patients were categorized into the primary and secondary infertility groups. No statistically significant differences in pregnancy outcomes were observed between these groups, even after PSM. Patients were also divided by maternal age at FET into those under 35 years and those aged 35–40 years. No significant differences in pregnancy outcomes were found between these age groups, both before and after PSM. Additionally, patients were classified based on EMT into two groups: 6–7 mm and 7–8 mm. Significant differences in clinical and biochemical pregnancy rates were observed between these groups, with results remaining significant after PSM (**Figures 1**, **2**).

## 4 Discussion

There are several reasons for utilizing FET, including the unsuitability of the endometrium during superovulatory cycles, the high risk of ovarian hyperstimulation syndrome (19), and improved cumulative live birth rates (20). One study noted an 82.5% increase in the number of FET cycles over eleven years, compared to a 3.1% increase in fresh embryo transfers (21). For patients with a TE, FET is preferred for ovulatory induction cycles due to concerns about the adequacy of EMT for implantation. Common endometrial preparation protocols include the NC and HRT protocols. The NC protocol is closely mimic natural pregnancy. The protocol is associated with high treatment compliance, simplicity, cost-effectiveness, and the production of endogenous estrogens that promote embryonic implantation. However, they require frequent ultrasound monitoring and have a higher cycle cancellation rate than HRT protocols (22). HRT protocols can stimulate endometrial growth through exogenous estrogen but incur higher costs due to the need for additional medication and associated financial and emotional burdens. Despite this, the current literature does not definitively address the optimal endometrial preparation protocol for FET. This study compared the NC and HRT protocols in patients with TE and found no significant differences in pregnancy outcomes, with results remaining consistent after PSM. Jie et al. (23) reported no difference in clinical pregnancy rates between natural and artificial cycles for patients with a TE, with clinical pregnancy rates of 52.1% and 44.4% in the HRT and NC groups, respectively, consistent with our findings.

Age significantly affects endometrial development. As women age, reduced endometrial blood flow and fewer estrogen and progesterone receptors lead to the decreased proliferation of endometrial glandular cells and blood vessels, resulting in a TE (18, 24). Steiner et al. identified age as an independent predictor of pregnancy outcomes (25). When analyzed by age group, we found no significant differences in pregnancy outcomes between protocols.

Several studies have shown that a TE is often associated with insufficient blood supply, glandular epithelial cell dysplasia, and reduced vascular endothelial growth factor expression (26, 27). However, there is no consensus on the definition of a TE. A meta-analysis of 1,170 studies found a significant reduction in ongoing pregnancy and live birth rates for patients with EMT ≤7 mm compared to those with EMT >7 mm (28). Liu et al. (29) found that clinical pregnancy and live birth rates declined with decreasing EMT in IVF and FET cycles. Most studies define an EMT of 7 mm or 8 mm on the day of trigger or ovulation as thin. This study compared patients with an EMT of 6–7 mm on the trigger day to those with an EMT of 7–8 mm on the day. The results showed that patients with an EMT of 6–7 mm had higher clinical and biochemical pregnancy rates with HRT protocol consistent even after adjusting for confounders in PSM. This study demonstrated that for patients with thin endometrium, if the EMT on trigger day was 6-7mm, the HRT protocol should be directly implemented in the subsequent FET cycles. The strategy not only significantly improves the clinical pregnancy rate but also effectively reduces,medical costs and alleviates the anxiety associated with prolonged infertility, thereby offering patients greater hope and confidence. Therefore, this study proposes that defining TE as EMT ≤ 7mm on the trigger day is a more rational and clinically relevant criterion. This definition enables attending physicians to initiate pharmacological or adjunctive therapies prior to the FETcycles to enhance endometrial thickness, thereby reducing the cancellation rate of cycles due to inadequate endometrial development and ultimately improving pregnancy success rates.

This study has several limitations. Although PSM balanced the baseline characteristics and adjusted for potential confounders, it did not eliminate the effects of other unknown confounders. The retrospective nature of the analysis, conducted at a single reproductive center where treatment regimens depend largely on physician experience, may introduce bias in protocol selection. Hence, multicenter prospective clinical trials are needed for more accurate results. Previous studies have shown a higher incidence of pregnancy complications with HRT cycles compared to NC cycles. However, this study did not statistically analyze the incidence of complications in patients with TE and different preparation regimens, which may affect the conclusions.

## 5 Conclusion

In conclusion, selecting an appropriate endometrial preparation protocol for patients with a TE is essential to optimize outcomes for both the mother and offspring in clinical practice. This selection should consider not only the EMT but also the associated costs and time requirements. For patients with an EMT of less than 7 mm on the trigger day, the HRT protocol is recommended for FET cycles.

## Data Availability

The datasets presented in this article are not readily available because The data is not publicly available due to their containing information that could compromise the privacy of research participants. Requests to access the datasets should be directed to Xiaoxue Ji, 15684622389@163.com.
